# Efficacy and safety of acupuncture for functional constipation: a randomised, sham-controlled pilot trial

**DOI:** 10.1186/s12906-018-2243-4

**Published:** 2018-06-15

**Authors:** Hye-Yoon Lee, Oh-Jin Kwon, Jung-Eun Kim, Mikyeong Kim, Ae-Ran Kim, Hyo-Ju Park, Jung-Hyo Cho, Joo-Hee Kim, Sun-Mi Choi

**Affiliations:** 1 National Clinical Research Center, Pusan National University Korean Medicine Hospital, Yangsan, Republic of Korea; 2 Clinical Research DivisionKorea Institute of Oriental Medicine, Daejeon, Republic of Korea; 3 Department of Internal Korean Medicine, Daejeon Oriental Hospital of Daejeon University, Daejeon, Republic of Korea; 4 Department of Acupuncture and Moxibustion Medicine, College of Korean Medicine, Sangji University, Wonju, Republic of Korea; 5 KM Standards Centre, Korea Institute of Oriental Medicine, Daejeon, Republic of Korea

**Keywords:** Constipation, Functional constipation, Acupuncture, Randomised controlled trial

## Abstract

**Background:**

The prevalence of functional constipation (FC) is 3–27%, and FC has been reported to cause discomfort in daily life and various complications. The treatment for FC depends on laxatives, and thus, effective and non-toxic alternative treatments are needed.

**Methods:**

We conducted a randomised, sham-controlled parallel-design, pilot trial. Participants with FC were randomly assigned to either the real acupuncture (RA) or sham acupuncture (SA) group. The RA consisted of eight fixed acupuncture points (bilateral ST25, ST27, BL52 and BL25) and four additional points targeted to the individual based on Traditional Korean medicine (TKM). SA consisted of shallow acupuncture insertion at 12 non-acupuncture points. Twelve sessions were provided over 4 weeks. The outcome measures were weekly defecation frequency (DF), spontaneous complete bowel movement (SCBM), Bristol stool scale (BSS) score and constipation assessment scale (CAS) score. The participants were followed for 4 weeks after the treatment.

**Results:**

Thirty participants were enrolled (15:15). The mean DF were 5.86 ± 5.62, 5.43 ± 3.39 and 5.79 ± 3.64 in the RA group and 3.73 ± 1.62, 5.00 ± 1.77 and 5.40 ± 1.96 in the SA group at weeks 1, 5, and 9, respectively. The increases in weekly SCBMs were 2.50 ± 3.86 and 2.71 ± 4.01 with RA and 2.33 ± 2.74 and 1.93 ± 2.25 with SA at weeks 5 and 9, respectively (mean difference [MD] 0.78). The BSS scores were 0.57 ± 1.72 and 1.09 ± 1.30 with RA and 0.15 ± 1.06 and 0.14 ± 0.88 with SA at weeks 5 and 9, respectively (MD 0.95). The CAS score changes were − 3.21 ± 2.91 and − 3.50 ± 3.98 with RA and − 2.67 + ±2.82 and − 2.87 ± 2.95 with SA at weeks 5 and 9, respectively. Greater improvements were observed in subgroup analysis of participants with hard stool. The numbers of participants who developed adverse events (AEs) were equal in both groups (four in each group), and the AEs were not directly related to the intervention.

**Conclusions:**

This clinical trial shows feasibility with minor modifications to the primary outcome measure and comparator. Acupuncture showed clinically meaningful improvements in terms of SCBMs occurring more than 3 times per week and in these improvements being maintained for 4 weeks after treatment completion. As this is a pilot trial, future studies are warranted to confirm the efficacy and safety.

**Trial registration:**

KCT0000926 (Registered on 14 November 2013).

**Electronic supplementary material:**

The online version of this article (10.1186/s12906-018-2243-4) contains supplementary material, which is available to authorized users.

## Background

Functional constipation (FC) refers to persistently difficult, infrequent or seemingly incomplete defecation without other organic diseases and without meeting the Irritable Bowel Syndrome criteria [1, 2]. The prevalence of FC varies according to diagnostic criteria and region. In South Korea, the self-reported constipation rate was 16.5% [3]. The prevalence of FC according to the Rome II criteria was reported to be 9.2% [3], which is relatively higher than that found in other Asian countries (3.0–7.2%) [4, 5]. The prevalences of FC have been reported to be 17.1% in Europe, 15.3% in Oceania and 1.9–27.2% in North America [6, 7]. Additionally, the female gender, an older age and a lifestyle of insufficient physical activity influence the prevalence of constipation [6–8].

Constipation patients experience decreases in health-related quality of life, and both patients and healthcare providers must manage the economic burden of this condition [9, 10]. One Korean study found that 42.9% of self-reported constipation patients (53.4% female, 20.4% male) feel discomfort during everyday life [3]. Furthermore, constipation can cause complications, including haemorrhoids, rectal prolapse, perineal descent, faecal impaction and stercoral ulcers [11].

Lifestyle changes and pharmacological or surgical treatments are used to manage constipation [1, 2, 12, 13]. Non-pharmacological biofeedback treatments have shown positive effects in several studies [12, 14, 15], but there are insufficient high-quality studies to draw definitive conclusions [16, 17]. Widely used pharmacological treatments include bulk-forming, osmotic and stimulant laxatives [1, 2, 12]. However, side effects, including colonic damage, exacerbated constipation, and melanosis coli, have been reported, particularly with the excessive use of stimulant laxatives [1, 13, 18]. Thus, these medications are recommended for short-term use when non-pharmacological interventions are ineffective [13], highlighting the need for acceptable, safe, timely and non-toxic alternative treatments.

Acupuncture is a treatment modality originating from traditional Chinese medicine. Acupuncture has been used to treat various digestive diseases [19, 20], and clinical trials have studied its application, along with electroacupuncture (EA), in constipation [21, 22]. A systematic review reported that acupuncture might be as effective as conventional treatments for chronic FC and colonic transit activity [23]. This review presented some limitations of existing studies, such as not using standard assessment tools and not having a placebo control in intervention studies.

Thus, we conducted a randomised, sham-controlled, parallel design pilot trial to explore the possible efficacy and safety of acupuncture for treating FC and assess the feasibility of performing a full-sized randomised controlled trial (RCT) in the future.

## Methods

### Study design

**Fig. 1:**
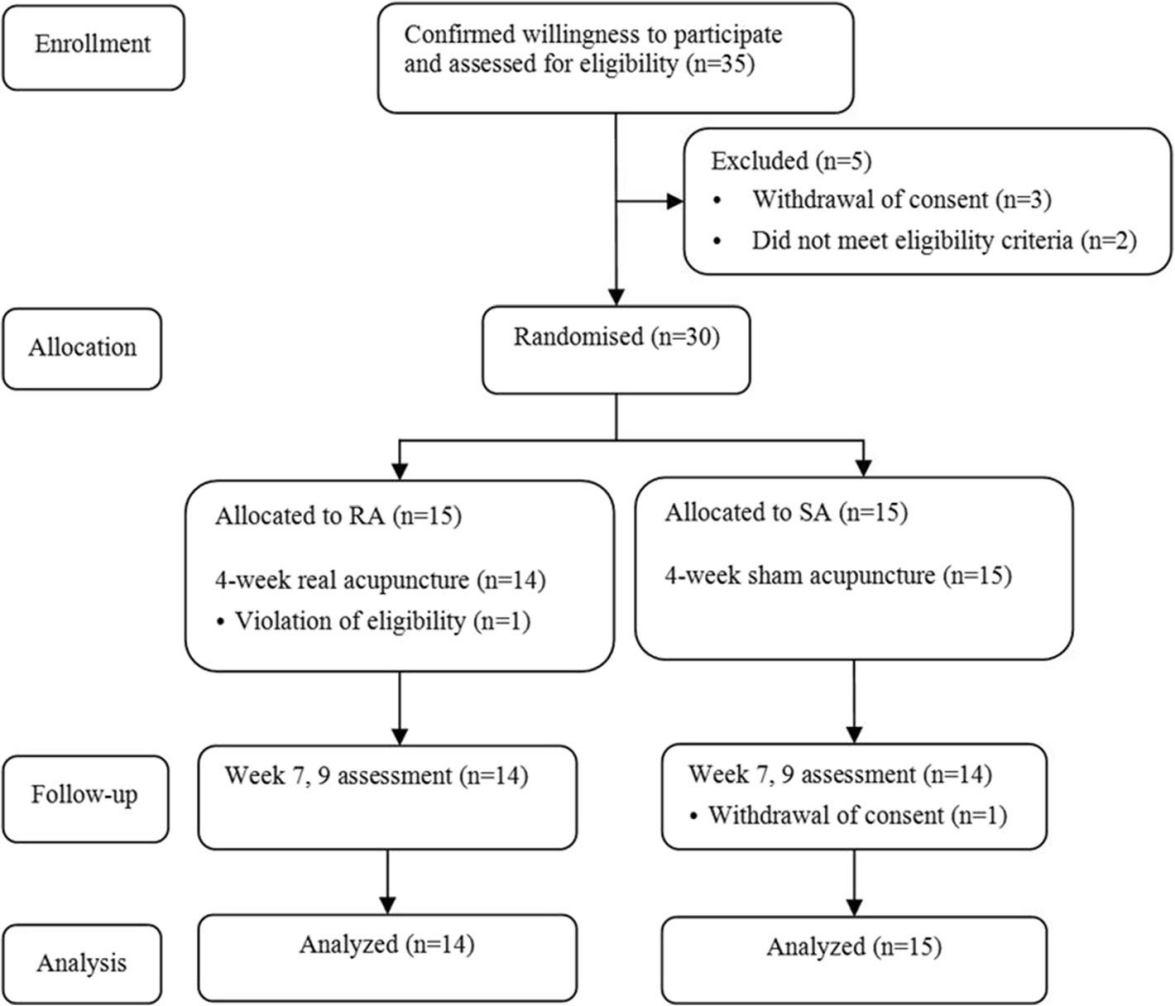
CONSORT flow chart of the study. RA, real acupuncture; SA, sham acupuncture

This was a randomised, sham-controlled, participant/assessor-blinded pilot trial conducted in a university hospital setting in South Korea from November 2013 to October 2015. Thirty participants with FC were registered and assigned to either the real acupuncture (RA) or sham acupuncture (SA) group; 15 participants were in each group. Twelve RA or SA sessions were performed over 4 weeks, and follow-up assessments were performed every 2 weeks for 4 weeks after intervention completion in each group. This study followed CONSORT guidelines (www.CONSORT-statement.org), and the CONSORT flow chart of the study is shown in Fig. 1.

### Participants

Eligible participants were aged between 19 and 65 years old, of either sex, and met the Rome III criteria for FC.

The exclusion criteria included having other causes of constipation, a history of bowel surgery or cholecystectomy, other severe medical diseases (e.g., insulin-dependent diabetic mellitus, heart, lung, liver or kidney disease, or malignancy), hypersensitivity reaction, being pregnant, expecting to be pregnant, actively nursing, receiving acupuncture, moxibustion, cupping or herbal medicine treatments for constipation within the past 4 weeks, or taking any medications or probiotics for constipation within the past 2 weeks.

Eligible participants who were willing to comply with the study protocol and provided written consent were asked to maintain a defecation diary for a one-week screening period. This diary was used by Korean medical doctors (KMDs) at visit 1 to confirm that the patients satisfied the Rome III criteria.

### Ethical considerations

This study was approved by the institutional review board (IRB) of the Daejeon Oriental Hospital, Daejeon University (djomc-112) and performed in compliance with the Declaration of Helsinki. All subjects were provided with verbal and written information on possible benefits, harms and the possible treatments for constipation other than the treatments offered in our clinical trial before signing the consent form. We obtained written informed consent from the subjects who decided to participate in the trial by their voluntary will. The safety assessment was performed at every visit by KMDs.

### Sample size

As this was a pilot clinical trial to explore the efficacy and safety of acupuncture for FC and assess trial feasibility, the sample size was not calculated to provide sufficient statistical power to support the hypothesis but was instead based on the number of participants available for enrolment and the minimum number of participants required for the purposes of a pilot study [24]. Accordingly, 12 participants were required for each group; considering a 20% withdrawal rate, we planned to enrol 30 participants.

### Randomisation

The 30 enrolled participants were randomly allotted into either the RA or SA group at a 1:1 ratio. The randomisation list was generated with a block-randomisation method using SAS® Version 9.1.3 (SAS Institute, Inc., Cary, NC, USA) by an independent statistician. The block size was randomly determined.

### Allocation concealment

After the randomisation list was created, it was coded, sealed in an envelope, and stored in a locked cabinet. The group allocations were sealed in opaque double envelopes and numbered in order according to the randomisation list. Upon participant enrolment, an envelope was opened to determine their group allocation. The KMDs in charge of the intervention verified the group allocations of the participants.

### Blinding

Assessors and participants were blinded. As practitioners cannot be blinded due to the nature of the intervention, they merely conducted acupuncture without unnecessary conversation or sharing information with other researchers. Assessors simply asked essential questions, which were specified in the case report form. Participants were scheduled to visit at different times to minimise both the exchange of information and the risk of bias.

### Intervention

Each group received 12 intervention sessions (3 times over 4 weeks). We used same acupuncture needles (0.25 mm in diameter and 40 mm in length, Donbgang Acupuncture Inc., Bundang, Sungnam, Republic of Korea), and conducted intervention and evaluation with the same number of times in same schedule.

**Fig. 2:**
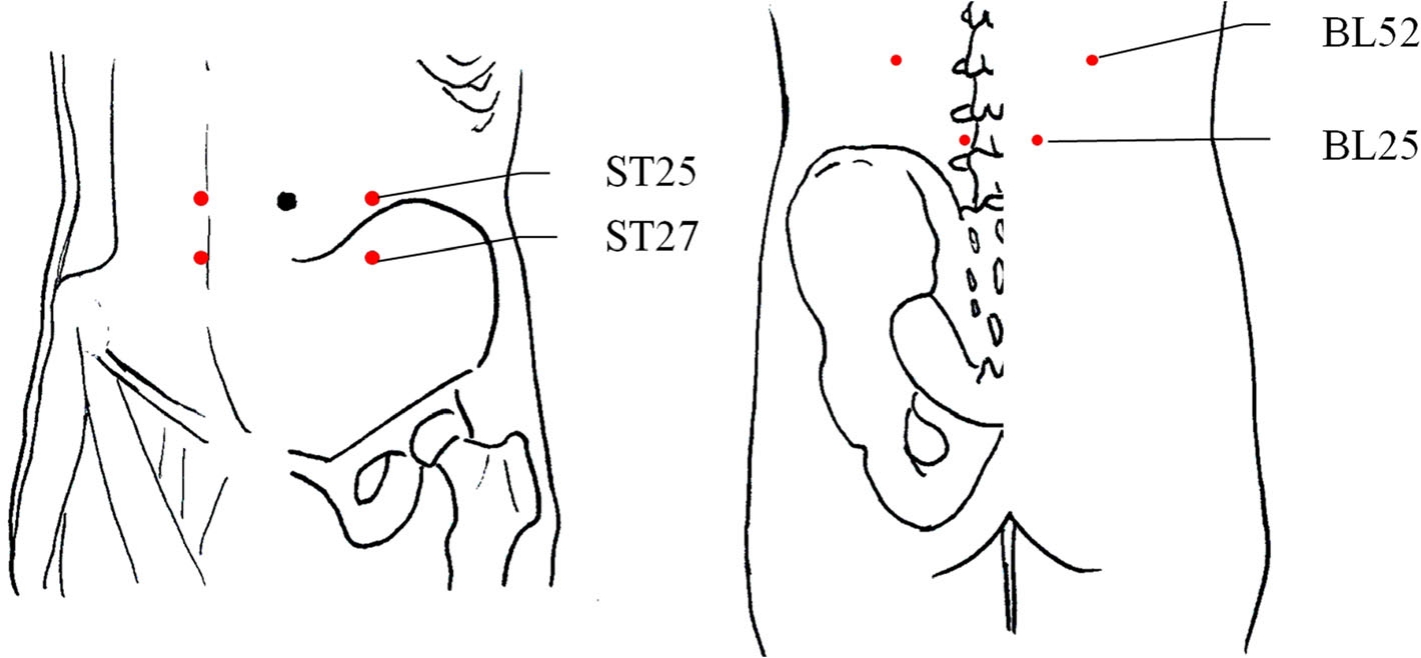
Acupuncture points of Real Acupuncture (RA)

The RA group received RA on eight fixed acupuncture points (bilateral ST25, ST27, BL52 and BL25) and four individualised acupuncture points. Sa-am acupuncture was used as the individualised acupuncture. The Sa-am acupuncture is a type of acupuncture based on traditional Korean medical (TKM) principles. The traditional theory about yin and yang, five element, and meridian are used in the application of Sa-am acupuncture. That is, five shu points of each meridian are used according to the creation and control cycles of five-element theory. Acupuncture points are selected based on the relationship of Governor, Mother and Son. For example, in case of deficiency of organ, governor point of its own meridian and of its governor meridian are sedated, while mother point of its own meridian and of its mother meridian are tonified. The set of these four acupuncture points is called ‘Jeong-Gyeok’. In case of excess of organ, the governor points should be tonified while son point of its own meridian and of its son meridian should be sedated. The set of these four acupuncture points is called ‘Seung-Gyeok’. In the present study, we used large intestine (LI) Jeong-Gyeok for LI deficiency, stomach (ST) Jeong-Gyeok for stomach deficiency, and liver (LR) Seung-Gyeok for liver excess [25–27]. The detailed location and procedures of RA can be found in Additional file 1 and Fig. 2. KMDs inserted the acupuncture needles using guide tubes, manipulated them (twitch, forward-and-backward) for de-qi, and maintained them for 30 min. Subsequently, the needles were again manipulated for de-qi and then removed. “de-qi” indicates that “sensory of soreness, numbness or tingling, fullness or pressure and heaviness” has occurred by proper acupuncture insertion [23, 28].

**Fig. 3:**
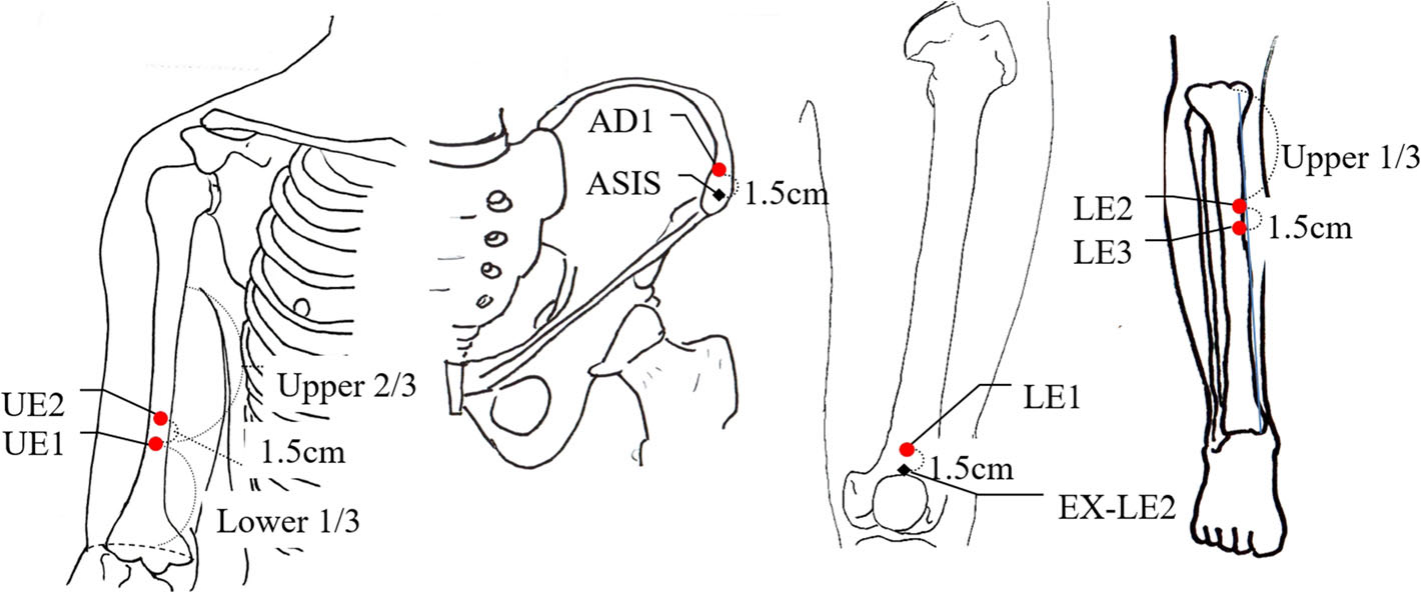
Points used for Sham Acupuncture (SA). UE, upper extremity, AD, abdomen; ASIS, anterior-superior iliac spine; LE, lower extremity; EX-LE2, *he ding*

The SA group received minimal acupuncture consisted of shallow acupuncture insertion (1–2 mm in depth) without manual stimulation or de-qi sensation on 12 non-acupuncture points for 30 min [29, 30]. The detailed location and procedures of SA can be found in Additional file 2 and Fig. 3. KMDs with over 4 years of clinical experience conducted the acupuncture procedures.

Each participant’s usual intake of dietary fibre drinks, coffee, sweet potatoes, or fermented milk was maintained. In the first screening stage, patients taking regular laxatives or had taken a laxative in the 2 weeks before enrollment were excluded according to our eligibility criteria, and subjects were not asked to discontinue their prescribed medication or change their standard medical care. All patients who participated in the present study were allowed to use laxative (rescue medicine) prescribed by the protocol when needed throughout the entire study period. We provided participants with Magnesium Hydroxide 500 mg as a rescue medication and had them take it if needed. The Magnesium Hydroxide has been recommended as effective treatment for constipation and widely used in clinical practice with agreement of more than 90% of Korean clinicians [31]. Each participant was asked to complete a daily diary recording the shape and frequency of defecation, related symptoms, and use of rescue medication. The number of days and amount of rescue medication taken by each participant were monitored and used for analysis of results. The intervention details have been described according to the Standards for Reporting Interventions in Clinical Trials of Acupuncture (STRICTA) criteria (Additional file 3).

### Outcomes

The weekly DFs, weekly spontaneous complete bowel movement (SCBMs) and mean Bristol stool scale (BSS) scores were recorded every week during the treatment period and the two follow-up visits. The constipation assessment scale (CAS) scores were evaluated at weeks 1, 3, 5, 7 and 9. Rescue medication use and AEs were investigated at each visit. The primary outcome was changes in DF at week 5; all others were secondary outcomes.

### Statistical analysis

Statistical analyses were performed using the full analysis set (FAS) population, which included a population as similar to intent-to-treat (ITT) as possible. All analyses were performed using SAS® Version 9.4 with a significance level of 5% and two-sided tests.

For the purpose of this pilot study, the analyses performed were mainly descriptive to present actual changes and treatment effects using the mean difference (MD) and Hedge’s g. The effect size was interpreted as follows: 0.2, small; 0.5, medium; 0.8, large; 1.2, very large; and 2.0, huge [32, 33].

Inter-group differences were examined using a Mixed-effect Model for Repeated Measures (MMRM). Paired-sample t-tests or Wilcoxon signed-rank tests were conducted to analyse the changes in each group after treatment. All AEs reported during the study period were charted, and the difference in the incidence of AEs between the two groups was assessed using the Chi-square test or Fisher’s exact test. The ‘last observation carried forward’ approach was used to replace the missing values, except in the MMRM. The MMRM method does not require an alternative step because it uses the maximum likelihood, which accounts for missing values.

A subgroup analysis was performed for participants with severe symptoms. DF and SCBM were relatively even in participants overall, while the BSS and CAS scores showed heterogeneity. Thus, severity was classified based on these two factors. Subgroup A included participants whose proportion of hard stool (BSS score 1–2) was > 25% of total defecation during one week, and subgroup B included participants whose CAS score was ≥7 at visit 1.

## Results

### Recruitment

Thirty-five participants were assessed for eligibility, but 5 (14.3%) were not enrolled (three withdrew consent; two did not meet the eligibility criteria).

Twenty-eight (93.3%) participants completed the trial. One participant in the RA group was excluded due to a violation of the inclusion criteria identified at visit 1, before any treatment was performed. One participant in the SA group withdrew consent.

The treatment-compliance rate, i.e., the percentage of treatment sessions participants actually received of all 12 planned sessions, was 99.4 ± 2.4% and 94.4 ± 19.3% in the RA and SA groups, with an overall rate of 96.8 ± 14.0%.

### Demographic characteristics

Both groups included more females than males, with 85.7 and 91.7% females in the RA and SA groups, respectively. The mean age (49.6 and 50.0 years old), height, body weight and body mass index were similar in both groups. Factors that may influence defecation, including dietary, exercise, smoking and drinking habits, were not significantly different between groups (Table 1).

**Table 1:** Baseline demographic and general characteristics

	RA	SA	*p*-value
Demographic characteristics			
Female	12 (85.7)	14 (91.7)	0.5977^a^
Age(years)	49.6 ± 12.7	50.0 ± 10.5	0.9348^b^
Height(cm)	159.2 ± 8.0	158.3 ± 5.8	0.7486^b^
Weight(kg)	61.5 ± 12.2	59.3 ± 9.6	0.5893^b^
BMI	21.1 ± 2.7	23.6 ± 3.1	0.6767^b^
Breakfast(times/week)			
≥ 5	5 (35.7)	10 (66.7)	0.2601^a^
3–4	6 (42.9)	4 (26.7)	
0–2	3 (21.4)	1 (6.7)	
Mealtime			
Regular	2 (14.3)	7 (46.7)	0.1086^a^
Irregular	12 (85.7)	8 (53.3)	
Whole-grain intake	3 (21.4)	2 (13.3)	0.6513^a^
Exercise	7 (50.0)	11 (73.3)	0.1956^c^
Smoking	2 (14.3)	0 (0.0)	0.2241^a^
Drinking	4 (28.6)	3 (20.0)	0.6817^a^
Past medication use	1 (7.1)	5 (33.3)	0.1686^a^

Data are presented as the mean ± standard deviation or frequency(%)

*RA* real acupuncture, *SA* sham acupuncture, *BMI* body mass index

^a^Fisher’s exact test

^b^independent two-sample t-test

^c^Chi-square test

### Primary outcome

After the four-week treatment period (week 5), DF decreased from 5.86 ± 5.61 to 5.43 ± 3.39 with RA, while it increased from 3.79 ± 1.62 to 5.00 ± 1.77 with SA. The MD was − 1.70 (− 3.94 to 0.55), Hedge’s g was 0.56, and the changes were not significantly different between the groups (*p* = 0.2933, Table 2).

**Table 2:** Effects of treatment with RA and SA by all participants, subgroup A, and subgroup B

			Baseline	Post-treatment^a^	Mean change	MD (95% CI), Hedge’s g	Follow-up^b^	Mean change	MD (95% CI), Hedge’s g
DF (freq/wk)	Total	RA (*n* = 14)	5.86 ± 5.62	5.43 ± 3.39	−0.43 ± 4.09	−1.70 (− 3.94, 0.55),	5.79 ± 3.64	− 0.07 ± 4.16	−1.74 (− 4.13, 0.66),
		SA (*n* = 15)	3.73 ± 1.62	5.00 ± 1.77	1.27 ± 1.10	0.56	5.40 ± 1.96	1.67 ± 1.72	0.53
	Subgroup A	RA (*n* = 5)	7.60 ± 9.40	6.60 ± 4.34	−1.00 ± 5.66	−1.75 (− 8.61, 5.11),	7.80 ± 4.66	0.20 ± 5.76	−0.30 (−7.34, 6.74),
		SA (*n* = 4)	4.00 ± 2.71	4.75 ± 2.36	0.75 ± 0.96	0.36	4.50 ± 1.73	0.50 ± 1.29	0.06
	Subgroup B	RA (*n* = 9)	7.00 ± 6.80	5.89 ± 3.86	−1.11 ± 4.94	−2.86 (−6.67, 0.94)	6.67 ± 4.09	−0.33 ± 5.12	−2.58 (− 6.70, 1.54),
		SA (*n* = 8)	3.25 ± 0.71	5.00 ± 1.51	1.75 ± 1.04	0.74	5.50 ± 2.27	2.25 ± 1.98	0.62
SCBM (freq/wk)	Total	RA (n = 14)	0.71 ± 1.20	3.21 ± 3.83	2.50 ± 3.86	0.17 (−2.37, 2.70),	3.43 ± 4.05	2.71 ± 4.01	0.78 (−1.67, 3.24),
		SA (n = 15)	1.13 ± 1.51	3.47 ± 2.45	2.33 ± 2.74	0.05	3.07 ± 2.19	1.93 ± 2.25	0.23
	Subgroup A	RA (n = 5)	0.60 ± 0.89	4.60 ± 5.18	4.00 ± 5.43	3.75 (−2.78, 10.28),	6.00 ± 5.43	5.40 ± 5.37	4.90 (1.56, 11.36),
		SA (n = 4)	2.00 ± 2.71	2.25 ± 2.63	0.25 ± 0.50	0.81	2.50 ± 2.52	0.50 ± 0.58	1.07
	Subgroup B	RA (n = 9)	0.56 ± 0.88	3.22 ± 4.41	2.67 ± 4.33	−0.21 (−3.88, 3.47),	3.44 ± 4.90	2.89 ± 4.83	0.76 (−3.29, 4.82),
		SA (n = 8)	0.63 ± 0.52	3.50 ± 2.07	2.88 ± 2.36	0.06	2.75 ± 2.31	2.13 ± 2.47	0.18
BSS	Total	RA (n = 14)	3.60 ± 1.09	4.17 ± 1.48	0.57 ± 1.72	0.42 (−0.66, 1.50),	4.69 ± 0.82	1.09 ± 1.30	0.95 (0.11, 1.79),
		SA (n = 15)	3.32 ± 1.38	3.47 ± 1.33	0.15 ± 1.06	0.29	3.46 ± 1.19	0.14 ± 0.88	0.84
	Subgroup A	RA (n = 5)	2.71 ± 0.64	4.68 ± 1.09	1.97 ± 1.23	1.06 (−0.64, 2.77),	4.60 ± 0.23	1.89 ± 0.51	1.19 (0.34, 2.03),
		SA (n = 4)	1.46 ± 0.42	2.36 ± 1.08	0.91 ± 0.82	0.87	2.16 ± 0.90	0.70 ± 0.56	2.00
	Subgroup B	RA (n = 9)	3.31 ± 1.12	4.08 ± 1.80	0.78 ± 2.11	0.96 (−0.89, 2.82)	5.01 ± 0.81	1.70 ± 1.10	1.91 (0.84, 2.98),
		SA (n = 8)	3.96 ± 1.31	3.77 ± 1.43	−0.19 ± 1.35	0.51	3.75 ± 1.15	−0.21 ± 0.95	1.76
CAS	Total	RA (n = 14)	7.93 ± 4.01	4.71 ± 3.73	−3.21 ± 2.91	−0.55 (−2.73, 1.64),	4.43 ± 3.98	−3.50 ± 3.98	−0.63 (−3.29, 2.02),
		SA (n = 15)	7.27 ± 3.01	4.60 ± 2.41	−2.67 + ±2.82	0.18	4.40 ± 2.85	−2.87 ± 2.95	0.17
	Subgroup A	RA (n = 5)	10.40 ± 1.82	5.40 ± 1.82	−5.00 ± 3.16	−3.50 (−8.90, 1.90),	4.20 ± 2.86	−6.20 ± 4.15	−4.70 (−11.10, 1.70),
		SA (n = 4)	6.25 ± 2.63	4.75 ± 1.50	−1.50 ± 3.70	0.92	4.75 ± 1.71	−1.50 ± 3.87	1.04
	Subgroup B	RA (n = 9)	10.56 ± 2.01	6.33 ± 3.67	−4.22 ± 3.03	−0.22 (−3.22, 2.77),	5.56 ± 4.36	−5.00 ± 3.84	−1.00 (− 4.71, 2.71),
		SA (n = 8)	9.63 ± 1.85	5.63 ± 2.00	−4.00 ± 2.73	0.08	5.63 ± 2.77	−4.00 ± 3.25	0.27

Data are presented as the mean ± standard deviation, mean difference (95% CI), or Hedge’s g.DF, defecation frequency

*SCBM* spontaneous complete bowel movement, *BSS* Bristol stool scale, *CAS* constipation assessment scale, *Subgroup A* participants whose proportion of hard stool (BSS type 1–2) > 25% of total defecation, *Subgroup B* participants whose CAS score ≥ 7, *RA* real acupuncture, *SA* sham acupuncture

^a^week 5

^b^week 9

### Secondary outcomes

#### Weekly DF

DF decreased by 0.07 ± 4.16 with RA but increased by 1.67 ± 1.72 with SA at week 9. The MD between the groups was − 1.74 (− 4.13 to 0.66) at week 9 (g = 0.53). There was no significant difference between the groups at any time-point (Table 2).

Four participants had a weekly DF > 7 (3 in the RA group and 1 in the SA group). When these participants were excluded, the weekly DF of the RA group was 3.82, 4.91, and 5.36 at weeks 1, 5 and 9, respectively and that of the SA group was 3.43, 4.79, and 5.29. Of patients with abnormally frequent defecation, the weekly DF in the RA group was 13.3, 7.3 and 7.3 at weeks 1, 5, and 9, respectively, (*n* = 3), while that in the SA group was 8, 8 and 7 (*n* = 1).

#### Weekly SCBM

RA showed an increase of 2.50 ± 3.83 at week 5 and 2.71 ± 4.01 at week 9, while SA showed corresponding increases of 2.33 ± 2.74 and 1.93 ± 2.25. Weekly changes within each group revealed a significant increase with RA from week 3 and a significant increase with SA from week 5 (*p* < 0.05). Inter-group comparisons did not reveal significant differences (g = 0.05 and 0.23 at weeks 5 and 9, respectively).

At weeks 5 and 9 in subgroup A, RA showed increases of 4.60 ± 5.18 and 5.40 ± 5.37, while SA showed increases of 2.25 ± 2.63 and 0.50 ± 0.58, revealing a large effect (g = 0.91 and 1.07) (Table 2).

#### Mean BSS score

The increase in BSS score was 0.57 ± 1.72 and 1.09 ± 1.30 at weeks 5 and 9 with RA compared to 0.15 ± 1.06 and 0.14 ± 0.88 in SA (g = 0.029 and 0.084, respectively). The change in the RA group was significant from week 7 (*p* < 0.05), but the inter-group analysis showed no significant difference.

At weeks 5 and 9 in subgroup A, RA showed increases of 1.97 ± 1.23 and 1.89 ± 0.51, while SA showed increases of 0.91 ± 1.82 and 0.70 ± 0.56 (g = 0.87 and 2.00, respectively). At weeks 5 and 9 in subgroup B, RA showed increases of 0.78 ± 2.11 and 1.70 ± 1.10, while SA showed decreases of 0.19 ± 1.35 and 0.21 ± 0.95 (g = 0.51 and 1.76, respectively) (Table 2).

#### CAS score

The CAS score in the RA group changed by − 3.21 ± 2.91 and − 3.50 ± 3.98 at weeks 5 and 9, while that in the SA group changed by − 2.67 ± 2.82 and − 2.87 ± 2.95, showing a very small effect between the groups (g = 0.18 and 0.17, respectively). Both groups showed significant improvements at each time-point compared to baseline (*p* < 0.05), and there was no significant difference between the groups.

In subgroup A, the CAS score in the RA group changed by − 5.00 ± 3.16 and − 6.20 ± 4.15 at weeks 5 and 9, while that in the SA group changed by − 1.50 ± 3.70 and − 1.50 ± 3.87, showing a large effect (g = 0.92 and 1.04, respectively).

#### Rescue medication

During the overall study period, 1 (7.1%) and 5 (33.3%) participants in the RA and SA groups needed to use the rescue medication. The number of days the rescue medication was used was 0.43 ± 1.60 in the RA group compared to 1.07 ± 2.43 in the SA group (Table 3).

**Table 3:** Rescue medication use during overall study period

	No. of participants*	*p*-value^a^	Days using rescue medication	*p*-value^b^
	*N* (%)		(mean ± SD)	
Total				
RA (*n* = 14)	1 (7.1%)	0.1686	0.43 ± 1.60	0.4154
SA (*n* = 15)	5 (33.3%)		1.07 ± 2.43	
Subgroup A				
RA (*n* = 5)	0 (0.0%)	0.1667	0.00 ± 0.00	0.2328
SA (*n* = 4)	2 (50.0%)		2.50 ± 4.36	
Subgroup B				
RA (*n* = 9)	1 (11.1%)	0.5765	0.67 ± 2.00	0.9615
SA (*n* = 8)	2 (25.0%)		0.63 ± 1.41	

Subgroup A, participants whose proportion of hard stool (BSS type 1–2) > 25% of total defecation; Subgroup B, participants whose CAS score ≥ 7

*RA* real acupuncture, *SA* sham acupuncture

*who used rescue medication during the period

^a^Fisher’s exact test

^b^independent two-sample t-test

### Safety

A total of 11 AE cases were reported over the course of 393 intervention sessions (2.8%), including 4 cases in the RA group (2.1%) and 7 cases in the SA group (3.5%). The number of participants who experienced AEs was the same in both groups (4 in the RA group and 4 in the SA group, *p* = 0.9087). Most AEs were mild or transient, with the exception of enteritis. The types of AEs were common cold, headache and insomnia in the RA group and common cold, headache, rhinitis, pain and enteritis in the SA group. No AEs were directly related to the interventions, and all AEs were completely cured within the trial period.

### Blind test

The blinding index scores [34] were 0.357 (95% confidence interval [CI] -0.068 to 0.782) for RA and − 0.500 (95% CI -0.883 to − 0.117) for SA, indicating that the blinding was adequate (*p* = 0.9999) (Table 4).

**Table 4:** Blind index test

Answers	RA (*n* = 14)	SA (*n* = 15)	*p*-value
RA (*n* (%))	8 (57.14%)	9 (64.29%)	0.9999
SA (*n* (%))	3 (21.43%)	2 (14.29%)	
Do not know (*n* (%))	3 (21.43%)	3 (21.43%)	
Blind index	0.357[−0.068, 0.782]	−0.500[− 0.883, − 0.117]	

pvalues determined by Fisher’s exact test

*RA* real acupuncture, *SA* sham acupuncture

## Discussion

As this study was not designed for statistical hypothesis verification, we evaluated effect size and clinical importance. The observed change in SCBMs is clinically meaningful, as an improvement ≥1 is usually considered a treatment success [35, 36]. In addition, the change was similar to [36] or larger [35, 37] than that of previous studies reporting significant improvements. The changes in BSS score were larger than those of a previous study, which revealed successful treatment by a 0.8 increase in the BSS score [38]. The change in the CAS score from > 7 to < 5 has clinical significance, as it indicates a change from severe constipation to constipation not requiring medical intervention [39, 40]; furthermore, score decreases > 3 can be considered significant [39]. In all, 7.1 and 33.3% of participants in the RA and SA groups, respectively, needed to use the rescue medication. The excessive use of laxatives is a known problem in Korea [18]. Thus, it would be meaningful for further clinical trials to investigate the medication-reduction effect of acupuncture. The baseline DF of the RA group was greater than that of subject groups in other constipation studies, which ranged from approximately 2.79 to 3.31 [35, 36]. As participants having abnormally frequent defecation were enrolled, increases in DF cannot be simply considered positive results. DF increased when those participants were excluded, but it decreased when they were included, suggesting the possibility that acupuncture exerts normalising effects on both abnormally increased and decreased DFs. While these effects cannot be determined in the present study, they merit future investigation.

Considering the specific effects of RA compared with SA, medium to large effects were observed with RA in terms of the BSS score (Table 2). In the subgroup analysis, participants having harder stools showed a large effect in the SCBMs and BSS and CAS scores, and participants having severe symptoms based on the CAS score showed a relatively larger effect on the BSS score. These findings suggest that acupuncture probably exerts non-specific effects on mild symptoms, while specific effects play an important role in refractory constipation. Furthermore, the effects of RA were maintained during the follow-up period, while those of SA decreased, showing significant differences in the BSS score and SCBMs in the latter period and a large effect size. In addition, RA showed an effect on SCBM more rapidly than did SA. Thus, the specific effects of acupuncture on bowel function are faster, larger and maintained for a longer period, whereas the non-specific effects are smaller and more temporary.

We considered the appropriateness of the comparator in terms of comparability with RA and blinding success. The blinding index revealed that participants in both groups tended to think they received RA. Thus, the attempt to blind participants seemed to be successful such that placebo effects, which reflect the psychological reactions of participants, were equally controlled [41]. However, SA showed clinically meaningful changes in some outcomes. Minimal acupuncture at non-acupuncture points has been reported to have moderate to large effects and induce some physiological effects comparable to those of RA [42–44]. A previous study on constipation reported no difference between deep and shallow insertion at the same acupuncture point, but no studies have used SA on non-acupuncture points [23]. Our results show that shallow insertion on non-acupuncture points is not an ideal comparator. Non-penetrating SA would be more appropriate in future studies.

For the purposes of a pilot study, we assessed trial feasibility. Using DF as the primary outcome might have resulted in false-negative results being overlooked because this study included participants with frequent defecation [12]. SCBM would be more adequate as a primary outcome because it considers both the frequency of defecation and symptoms of discomfort, such as a sensation of incomplete evacuation, anorectal obstruction, or excessive straining. To determine specific effects on certain measures, such as frequency or hardness, the eligibility criteria should be revised to include only specific types of constipation.

In participants who had hard stool (subgroup A), RA showed larger effects on SCBM and the BSS and CAS scores compared with those observed in the overall participant population and subgroup B. Accordingly, participants having hard stools could be grouped as potential responders, meriting further studies.

The recruitment rate of screened participants, compliance rate and completion rate were acceptable. However, if future studies target participants with severe symptoms, which seemed to be a potential response group in this study, the recruitment rate may be slower; thus, proper strategies are needed. Future studies would be feasible with some modifications to the primary outcome and comparator.

A sample size calculation for a future full-sized RCT was conducted based on SCBM. A recent study revealed that sham EA showed a change in SCBM of 0.87 (0.73 to 0.97) [37]. Supposing a change in SCBM of 0.87 resulting from inert SA and adopting the common SD of 3.33 and a change in RA of 2.5, 66 participants per group would be required, without considering a drop-out rate. Targeting participants with hard stool and adopting the common SD of 4.12 and a change in RA of 4.0, 28 participants per group would be required.

Regarding safety, 2.1 and 3.5% AEs were reported in the RA and SA groups, respectively, none of which were deemed to be related to the interventions over the course of all treatment sessions. These results corresponded with those of previous studies reporting acupuncture as safe with a low risk of accidents [45, 46]. The acupuncture procedure used in our study also seemed to be safe, but more data should be accumulated for confirmation.

The treatment mechanism could not be studied in this clinical trial, but some possible mechanisms have been reported in previous studies. ST25 was reported to improve interstitial Cajal cell expression and to recover colonic smooth muscle atrophy in rats with slow transit constipation [35]. EA on abdominal acupuncture points, including ST25, enhanced parasympathetic nerve activity in female constipation patients [47]. BL25 stimulation significantly rescued inhibited jejunal motility amplitude [48]. The observed effects of acupuncture might be related to these results, but further studies are needed to elucidate the mechanism of action.

Our study has strengths and weakness, as discussed below. This clinical trial has significance in that it set a sham-intervention control group to evaluate the efficacy of manual acupuncture in FC. The strengths of this study are as follows: it was previously registered on a clinical trial registry to minimise selective-reporting bias; participant blinding was successfully achieved using SA; and validated assessment tools were used, the lack of which has been a noted limitation of previous acupuncture studies. However, this study also has some limitations. First, we included few participants, which might lead to false-negative results. The results of our study cannot be generalised to support the efficacy and safety of acupuncture for FC because the sample size calculation for hypothesis acceptance was not conducted for this pilot trial. Shallow needle insertion in non-acupuncture points showed clinically meaningful changes, which made it difficult to distinguish the specific effect of RA, and careful interpretation was required. Finally, an FAS analysis was conducted instead of an ITT analysis because no data were obtained from one participant after randomisation. If an ITT analysis was conducted with complete follow-up data, it might have yielded different results.

## Conclusion

This study demonstrated that a future clinical trial would be feasible with some modifications to the primary outcome measure and comparator. An additionally finding is that twelve acupuncture sessions over 4 weeks have possible effects of increasing stool consistency and SCBM, particularly in participants with severe symptoms. In the future, a full-sized randomised controlled trial with a long-term follow-up period should be conducted to confirm these efficacy and safety findings.

## Additional files

Additional file 1: Details of Real Acupuncture. (DOCX 28 kb)

Additional file 2: Details of Sham Acupuncture. (DOC 36 kb)

Additional file 3: Checklist for items in STRICTA 2010. (DOCX 18 kb)

## Abbreviations

AE: Adverse event
BSS: Bristol stool scale
CAS: Constipation assessment scale
DF: Defecation frequency
FAS: Full analysis set
FC: Functional constipation
IRB: Institutional Review Board
ITT: Intent-to-treat
KMD: Korean medical doctor
MD: Mean difference
MMRM: Mixed-effect model for repeated measures
RA: Real acupuncture
RCT: Randomised controlled trial
SA: Sham acupuncture
SCBM: Spontaneous complete bowel movement
TKM: Traditional Korean medicine
