# Supplementary material for: Efficacy and safety of acupuncture for functional constipation: a randomised, sham-controlled pilot trial
Source: BMC Complement Altern Med. 2018 Jun 15;18:186. doi: 10.1186/s12906-018-2243-4 (PMC6002973; doi:10.1186/s12906-018-2243-4)
Supplement: Supplementary file 1 — Details of Real Acupuncture. (DOCX 28 kb) [file 12906_2018_2243_MOESM1_ESM.docx]

**Additional file 1. Details of Real Acupuncture**

| **Detail** | **Contents** | | | | | | | | | |
| --- | --- | --- | --- | --- | --- | --- | --- | --- | --- | --- |
| Number of needle insertions per subject per session | 12 (8 fixed points and 4 individualized points) | | | | | | | | | |
| Names and location^a^ of  fixed points (bilateral) | - ST25: 6 cm lateral to the centre of the umbilicus - ST27: 6 cm inferior to the centre of the umbilicus, 6 cm lateral to the anterior median line. - BL25: at the same level as the inferior border of the spinous process of the fourth lumbar vertebra (L4), 4.5 cm lateral to the posterior median line. - BL52: at the same level as the inferior border of the spinous process of the second lumbar vertebra (L2), 9 cm lateral to the posterior median line | | | | | | | | | |
| Depth of insertion of  fixed points | - ST25, ST27: 3-4.5 cm   - BL25, BL52: 1.5-3cm | | | | | | | | | |
| Names of individualized points (unilateral) |  | | Pattern | Acupuncture points | | Tonification | | Sedation | |  |
|  |  | | Deficiency of LI | Jeong-Gyeok^b^ of LI | | SP3, LU9 | | HT8, LU10 | |  |
|  |  | | Deficiency of ST | Jeong-Gyeok^b^ of ST | | SI5, ST41 | | GB41, ST43 | |  |
|  |  | | Excess of LR | Seung-Gyeok^c^ of LR | | LU8, LR4 | | ST8, LR2 | |  |
| Locations^a^ of  Jeong-Gyeok^b^ of LI | - SP3: in the depression proximal to the 1st metatarsophalangeal joint, at the border between the red and white flesh - LU9: between the radial styloid process and the scaphoid bone, in the depression ulnar to the abductor pollicis longus tendon - HT8: in the depression between the fourth and fifth metacarpal bones, proximal to the fifth metacarpophalangeal joint - LU10: radial to the midpoint of the first metacarpal bone, at the border between the red and white flesh | | | | | | | | | |
| Locations^a^ of  Jeong-Gyeok^b^ of ST | - SI5: in the depression between the triquetrum bone and the ulnar styloid process - ST41: in the depression at the centre of the front surface of the ankle joint, between the tendons of extensor hallucis longus and extensor digitorum longus - GB41: distal to the junction of the bases of the fourth and fifth metatarsal bones, in the depression lateral to the fifth extensor digitorum longus tendon - ST43: between the second and third metatarsal bones, in the depression proximal to the second metatarsophalangeal joint. | | | | | | | | | |
| Locations^a^ of  Seung-Gyeok^c^ of LR | - LU8: between the radial styloid process and the radial artery, 3 cm superior to the palmar wrist crease. - LR4: in the depression medial to the tibialis anterior tendon, anterior to the medial malleolus. - HT8: Between the fourth and fifth metacarpal bones, where the tip of the little finger rests when a fist is made - LR2: between the 1st and 2nd toes, proximal to the web margin, at the border between the red and white flesh. | | | | | | | | | |
| Depth of insertion of individualized points |  | Jeong-Gyeok^b^ of LI | | | Jeong-Gyeok^b^ of ST | | Seung-Gyeok^c^ of LR | |  |  |
|  |  | SP3: 0.9-1.5 cm  LU9: 0.6-0.9 cm  HT8: 0.9-1.5 cm  LU10: 0.9-1.5 cm | | | SI5: 0.6-1.2 cm  ST41: 0.9-2.4 cm  GB41: 0.9-1.5 cm  ST43: 0.9-1.5 cm | | LU8: 0.6-0.9 cm  LR4: 0.9-1.5 cm  HT8: 0.9-1.5 cm  LR2: 0.6-0.9 ccm | |  |  |
| Response sought | De-qi | | | | | | | | | |
| Needle stimulation | Manual stimulation (twitch, forward-and-backward),  twice per treatment (after insertion and before removal) | | | | | | | | | |
| Needle retention time | 30 minutes | | | | | | | | | |
| Needle type | Sterile steel acupuncture needles 0.25 mm in diameter and 40 mm in length (Donbgang Acupuncture Inc., Bundang, Sungnam, Republic of Korea) | | | | | | | | | |
| Number of treatment sessions | 12 sessions | | | | | | | | | |
| Frequency and duration of treatment sessions | 3 times over 4 weeks | | | | | | | | | |

^a^, according to Korean Medicine Convergence Research Information Center (KMCRIC, <https://www.kmcric.com>); LI, large intestine; ST, stomach; LR, liver; ^b^, Jeong-Gyeok is a Korean pronunciation meaning the set of acupuncture points for tonifying the deficient organ; ^c^, Seung-gyeok is a Korean pronunciation meaning the set of acupuncture points for sedating the excessive organ.
