# Supplementary material for: Efficacy and safety of acupuncture for functional constipation: a randomised, sham-controlled pilot trial
Source: BMC Complement Altern Med. 2018 Jun 15;18:186. doi: 10.1186/s12906-018-2243-4 (PMC6002973; doi:10.1186/s12906-018-2243-4)
Supplement: Supplementary file 2 — Details of Sham Acupuncture. (DOC 36 kb) [file 12906_2018_2243_MOESM2_ESM.doc]

**Additional file 2. Details of Sham Acupuncture**

| **Detail** | **Contents** |
| --- | --- |
| Number of needle insertions per subject per session | 12 |
| Names and locations of points used | Names:   - Non-acupuncture points of arms, abdomen and legs - Bilateral UE1, UE2, AD1, LE1, LE2, LE3   Locations:   - UE1: upper 2/3 of the connecting line of anterior axillary fold and cubital crease, between Pericardium Meridian and Lung Meridian - UE2: 1.5 cm above UE1 - AD1: 1.5 cm above ASIS - LE1: 1.5 cm above EX-LE2 (*he ding*) - LE2: upper 1/3 of medial part of tibia - LE3: 1.5 cm below LE2 |
| Depth of insertion (based on a specified unit of measurement or on a particular tissue level) | 1-2 mm |
| Response sought | None |
| Needle stimulation | None |
| Needle retention time | 30 minutes |
| Needle type | Sterile steel acupuncture needles 0.25 mm in diameter and 40 mm in length (Donbgang Acupuncture Inc., Bundang, Sungnam, Republic of Korea) |
| Number of treatment sessions | 12 sessions |
| Frequency and duration of treatment sessions | 3 times over 4 weeks |

UE, upper extremity; AD, abdomen; ASIS, anterior superior iliac spine; LE, lower extremity; TKM, traditional Korean medicine; KMD, Korean medical doctor.
