# Supplementary material for: Efficacy and safety of acupuncture for functional constipation: a randomised, sham-controlled pilot trial
Source: BMC Complement Altern Med. 2018 Jun 15;18:186. doi: 10.1186/s12906-018-2243-4 (PMC6002973; doi:10.1186/s12906-018-2243-4)
Supplement: Supplementary file 3 — Checklist for items in STRICTA 2010. (DOCX 18 kb) [file 12906_2018_2243_MOESM3_ESM.docx]

**Additional file 3. Checklist for items in STRICTA 2010**

| **Item** | **Detail** | **Page number** |
| --- | --- | --- |
| **1. Acupuncture rationale** | 1a) Style of acupuncture (e.g. Traditional Chinese Medicine, Japanese, Korean, Western medical, Five Element, ear acupuncture, etc) | 7 |
|  | 1b) Reasoning for treatment provided, based on historical context, literature sources, and/or consensus methods, with references where appropriate | 3, 4, 7, 16 |
|  | 1c) Extent to which treatment was varied | 7 |
| **2. Details of needling** | 2a) Number of needle insertions per subject per session (mean and range where relevant) | 7, additional file 2 |
|  | 2b) Names (or location if no standard name) of points used (uni/bilateral) | 7, additional file 2, figure 2 |
|  | 2c) Depth of insertion, based on a specified unit of measurement, or on a particular tissue level | 7,8  additional file 2 |
|  | 2d) Response sought (e.g. *de qi* or muscle twitch response) |  |
|  | 2e) Needle stimulation (e.g. manual, electrical) |  |
|  | 2f) Needle retention time |  |
|  | 2g) Needle type (diameter, length, and manufacturer or material) |  |
| **3. Treatment regimen** | 3a) Number of treatment sessions |  |
|  | 3b) Frequency and duration of treatment sessions |  |
| **4. Other components of treatment** | 4a) Details of other interventions administered to the acupuncture group (e.g. moxibustion, cupping, herbs, exercises, lifestyle advice) | 8 |
|  | 4b) Setting and context of treatment, including instructions to practitioners, and information and explanations to patients | 7,8 |
| **5. Practitioner background** | 5) Description of participating acupuncturists (qualification or professional affiliation, years in acupuncture practice, other relevant experience) | 8 |
| **6. Control or comparator interventions** | 6a) Rationale for the control or comparator in the context of the research question, with sources that justify this choice | 15 |
|  | 6b) Precise description of the control or comparator. If sham acupuncture or any other type of acupuncture-like control is used, provide details as for Items 1 to 3 above. | 8, additional file 3, figure 3 |
